# Tailoring rice varieties to consumer preferences induced by cultural and colonial heritage: Lessons from New Rice for Africa (NERICA) in The Gambia

**DOI:** 10.1177/00307270211019758

**Published:** 2021-05-26

**Authors:** Kofi Britwum, Matty Demont

**Affiliations:** 1 School of Business and Professional Studies, Upper Iowa University, Fayette, IA, USA; 2 International Rice Research Institute (IRRI), Los Baños, Laguna, Philippines

**Keywords:** Product profile, breeding, framed field experiment, double hurdle model, rice quality, labeling

## Abstract

Rice breeding priorities in Africa often focus on agronomic gains. However, being a net importer of rice, the continent’s varietal replacement success also crucially hinges on new varieties’ market competitiveness vis-à-vis imports. Markets have been profoundly shaped by cultural and colonial heritage. Indigenous preferences for African rice can be traced back to ancient rice domestication and have been subsequently influenced by Asian rice import standards as a result of colonial import substitution policies. New Rice for Africa (NERICA) crosses between African and Asian rice species have the potential to reconcile these dual indigenous/import preferences, but little is known about their market competitiveness. We use auction market data to assess the intrinsic and extrinsic consumer value of NERICA in The Gambia relative to two market standards: branded, Asian rice imports and the most popular locally grown Asian rice variety. We categorize rice consumers into four market segments, based on their heritage as evidenced by their preferences and genealogical lineages. NERICA outperforms both Asian rice standards in terms of market competitiveness, and its value is further reinforced by colonial heritage and labeling, but somewhat weakened by cultural heritage. Consumers were found to pay price premiums for NERICA in the range of 5% to 22% relative to Asian rice imports, with the highest premiums offered by consumers with colonial heritage, representing 86% of the sample. Maintaining and expanding this market will require breeders to incorporate trait mixes that reconcile agronomic gains and consumer preferences induced by cultural and colonial heritage.

## Introduction

Rice has become an important staple in sub-Saharan Africa (SSA), displacing many coarse grains such as sorghum and millet, and tubers (Nigatu et al., 2017). However, rice production in SSA is severely challenged with productivity issues and, as a result, the region has become increasingly dependent on imports. For example, during the decade 2009–2019, SSA annually imported 12.4 million tons of milled rice, 8.2 million destined for West Africa (Soullier et al., 2020). Countries along coastal belts with major seaports feature huge consumption poles and urban markets for rice, which are usually predominantly served by rice imports. With the aim of reducing import dependency, rice breeding priorities in Africa often focus on agronomic gains. However, import dependency also implies that varietal replacement programs should not only focus on replacing obsolete, underperforming, locally grown varieties, but also on substituting the competing product, i.e. rice imports, usually of Asian origin.

As part of efforts to encourage local production of the continent’s staple, the Coalition of African Rice Development (CARD) is currently revising the National Rice Development Strategies (NRDS, 2014) of 32 SSA countries with the aim of doubling rice production from 28 million to 56 million MT in 2030 (CARD, 2021). An important piece of the NRDS has been the promotion and expansion of New Rice for Africa (NERICA) varieties. Bred as a hybrid between the original African rice species *O. glaberrima* and the Asian variety *O. sativa*, NERICA blends traits from both, boasting important agronomic benefits such as weed, biotic and abiotic stress resistance, and higher yields (Wopereis et al., 2008), with potential yield of some NERICA varieties as high as 6 to 7 tons per hectare (Africa Rice, 2020). Notwithstanding the agronomic genetic gains of NERICA, the variety, and other local varieties can only meaningfully contribute to the NRDS goals of doubling rice production if agronomic breeding priorities are aligned with preferences downstream, that is, the ultimate consumer, and if local varieties can compete price- and quality-wise with competing rice products in import-biased markets (Demont et al., 2017).

Not much, however, is known about African consumers’ preferences for NERICAs and how these varieties compete against imports in urban markets. Even less is known about how cultural and colonial heritage affect NERICAs’ competitiveness in these markets. According to the cultural heritage hypothesis initially introduced by Demont (2013) and validated by Demont and Ndour (2015) and Demont et al. (2017), there is evidence that consumers in geographical proximity to primary and secondary centers of African rice domestication, or with genealogical lineages tracing back to the original rice domesticators tend to prefer and place price premiums on local rice over imported versions, reflecting trait preferences for African rice relative to Asian imports. The introduction of the NERICA variety makes the examination of these issues even more compelling; while the variety was bred to mainly capture superior agronomic traits in the African and Asian species, it allows us to explore whether this overarching goal of superior genetics unwittingly reconciled the dichotomy of consumer preferences for indigenous African rice versus Asian imports induced by cultural and colonial heritage.

The Gambian rice sector provides an ideal setting for studying the demand for NERICA. According to one historical account, the African rice species, *Oryza glaberrima*, was domesticated in present-day Mali, but spread to other centers of secondary domestication including the coast of The Gambia, Senegal’s Casamance region, and Guinea Bissau (Linares, 2002). Endowed with a 3,000-year-old cultural heritage, the Gambian rice sector has subsequently been exposed to century-long colonial import substitution policies, leading to massive influx of broken Asian rice (Moseley et al., 2010). The goal of this study was thus to examine market competitiveness of NERICA of African-Asian origin in this setting through a framed field experiment. Specifically, the study sought to determine the influence of indigenous preferences shaped by lineages to original rice domesticators on the one hand (cultural heritage), and preferences induced by decades-long rice imports on the other (colonial heritage), on urban Gambian consumers’ willingness to pay (WTP) for African-Asian NERICA relative to two market standards: branded, Asian rice imports and the most popular locally grown Asian rice variety. Secondary goals of the study were to evaluate the influence of intrinsic and extrinsic characteristics of NERICA on preferences for them, and to determine the influence of sensory experience on preferences for NERICA. To the best of our knowledge, this is the first study to explicitly examine linkages between consumer preferences for a staple and cultural and colonial heritage. In the next section, we expand the cultural heritage hypothesis further, focusing on the case of The Gambia.

## The Gambian rice sector’s cultural and colonial heritage

Domestication of the African rice species is thought to have originated in the Inner Delta region of the Niger River, from where it spread downstream along The Gambia and Senegal rivers (Linares, 2002; Sharma, 2010). Secondary centers of rice domestication boast a 3,000-year-old history, with two ethnic groups, the *Mandé* and *Jola* (known as *Diola* in French) believed to have been among early rice domesticators. Historical accounts note that the *Mandé* people lived in the Upper Niger Basin in the 13th century, and gradually migrated south and south-west to regions that now stretch from The Gambia to Ivory Coast (Sharma, 2010). The *Jola* people, on the other hand, settled along the Casamance River (of Senegal) in the 16th century, which is north of the Gambian River. Consequently, some ethnic groups in The Gambia have lineages that can be traced back to these original rice domesticators, also indicated in this study as being endowed with rice *cultural heritage*.

According to the *cultural heritage hypothesis*, indigenous preferences for local rice are fundamentally shaped by consumers’ cultural ties to early rice domesticators. In a comprehensive study of 15 West African countries including The Gambia, Soullier et al. (2020) explained that while areas with rice cultural heritage are somewhat shielded from competitive pressure from the world market thanks to strong local rice preferences, this comparative advantage in demand for local rice unfortunately tends to slow down competitive investment reactions in value chain upgrading such as in modern, industrial and semi-industrial milling technologies.

In The Gambia, the patchwork of agricultural policies from the 1960s has led to a divide between urban and rural consumers. Moseley et al. (2010) captured this as the “emergence of two Gambias.” They described that urbanized areas along the country’s coastal seaboard have become receptacles for relatively inferior imported broken rice from Asia, while the other “Gambia” lives in the interior regions where rice is cultivated, albeit not in substantial volumes to enable supply to urbanized areas. Similar to their northern neighbors in Senegal and Mauritania, years-long importation of broken rice from Indochina has altered preferences toward broken rice in The Gambia, while local rice fetches price premiums over imported rice (Brüntrup et al., 2006; John, 2015). Thus, broken grains cannot be viewed as an indigenous rice trait, but rather, an imported/colonial characteristic. Although broken rice is considered a by-product in Asia, in the Senegambia, the colonial administration introduced broken rice as a cheaper and easier-to-prepare substitute for millet in the traditional millet-based fish dishes. The 100% broken standard grade mimics the grain shape of millet and outperforms unbroken rice in terms of texture and sauce absorption in the resulting popular Gambian dish *benachin*, which translates into “one pot,” reflecting its preparation method of mixing broken rice, fish and other ingredients in a single cooking pot (somewhat like paella) (Dieng, 2012). Hence, similar to trends in South Asia (Custodio et al., 2019; Mottaleb and Mishra, 2016; Mottaleb et al., 2017), demand for broken rice in The Gambia reflects a derived demand for fine grain texture, driven by popular rice-based dishes. Other premium traits associated with colonial heritage include preferences for fragrance, and a higher swelling capacity (as a result of imported rice typically being stored for a longer period of time). Alternatively, a key indigenous rice trait is unbroken, non-fragrant grains, which were observed to be the preferred grain characteristics among traditional *Jola* women who live south of Senegal’s Casamance River (Linares, 2002).

Notably, the “two Gambias” (Moseley et al., 2010) have evolved from exposure to both cultural and colonial heritage with a dichotomy of rice consumer preferences for either (i) African traits (unbroken grains, no fragrance, *O. glaberrima* parentage) induced by indigenous cultural heritage, on the one hand, and (ii) Asian traits (broken grains, fragrance, high swelling capacity, *O. sativa* parentage) induced by colonial heritage as a result of import substitution policies, on the other. While satisfying these dual trait preferences could be challenging for rice breeders, NERICA varieties, which blend characteristics between African and Asian rice have the unique potential to reconcile these dual preferences. Originally introduced to The Gambia in 1998 on a participatory varietal selection basis (Dibba et al., 2012), NERICA adoption is reportedly widespread, in fact, in all six agricultural regions (Dibba et al., 2017), with adoption rates among rice farmers estimated to be about 66% (Dibba et al., 2015). Beyond the agronomic advantages of NERICA and other local varieties, their success in the market may hinge on their appeal to the consumer segments that have been shaped by cultural and colonial heritage.

## Experimental procedures

### Sampling

Framed field experiments based on auction markets were conducted in July 2010, targeting women 18 years and older in and around the central market of Serrekunda. A total of 10 experimental sessions with 10 participants each were conducted over 5 days, each day featuring a session in the morning and in the afternoon. Trained research assistants facilitated participant recruitment, and randomly approached women market goers about participating in a market research study for one and a half hours. To compensate participants for their time, they were told they would earn a gift of up to 162 Gambian Dalasi (GMD162, approximately US$5.30), a biscuit (cookie), and one kilogram of rice. Those who consented were transported to a cinema hall located a short distance away from the Serrakunda market. Given fixed seating in the hall, the experiments were all conducted on stage. A total of 100 women participated in the experimental sessions over the 5-day period.

### Products

**Figure 1.**
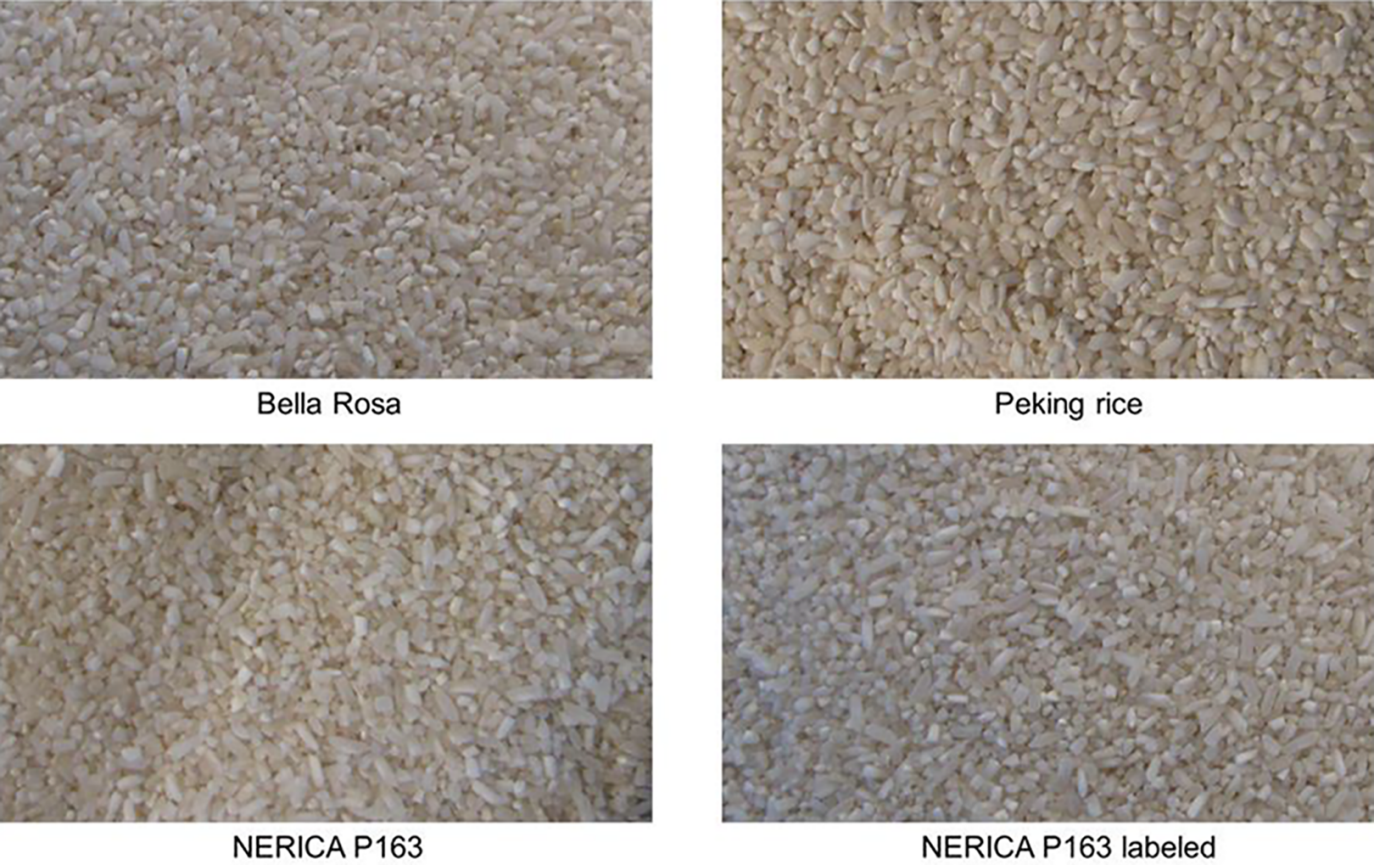
The four rice products used in the experimental auctions.

The auction experiments involved three different rice varieties: a popularly known imported and cheap brand, *Bella Rosa*, commonly available in urban markets, a locally grown variety of Asian *O. Sativa* origin, Peking, and a local NERICA variety WAB450-I-B-P163-4-1, which is commonly known as P163 (see Figure 1). As The Gambia had widely promoted NERICA since its arrival, in developing the auctions, an important objective in the design was to examine the impact of branding through a NERICA label on purchase intentions and preferences for local rice. Toward this goal, two versions of the same NERICA variety were presented in 50 kg bags on a table in front of the participants, one labeled, and the second unlabeled to capture the intrinsic and extrinsic value of NERICA. The imported rice brand, *Bella Rosa*, was designated the benchmark against which the three other rice types were measured. The NERICA variety was thus tested against two Asian market standards, the designated *Bella Rosa* benchmark, an Asian import, and the locally grown Asian variety, Peking. Hence, the auction tested four products: (i) branded imported *Bella Rosa* (benchmark); (ii) labeled NERICA; (iii) unlabeled NERICA (anonymous); and (iv) Peking (anonymous).

### Experimental auctions

We followed Roosen et al. (1998) by first endowing participants with a kilogram of the imported *Bella Rosa*, and subsequently requested their willingness to upgrade, and then their WTP to upgrade *Bella Rosa* into the alternative rice products. The Vickrey (1961) second price auction mechanism was used to elicit WTP bids due to its incentive compatible properties. According to the workings of the second price auction, the highest bidder receives the item, but pays the bid of the second highest bidder. Prior to the rice auctions, a practice round was conducted to familiarize participants with the experimental process. The trial session involved three well-known brands of biscuits (cookies). Here, participants were endowed with the cheaper Lolo biscuit, and requested to bid amounts to exchange this for the more expensive Sixer and Ginger biscuits. Given the diverse ethnicities of participants, the auctions were explained in three different languages, *Wolof, Pulaar,* and *Mandinka*, languages commonly spoken in the area.

After the research team were assured participants had understood the auction procedure, and after answering questions, each participant was presented with one kilogram of the four rice varieties placed in a triangular fashion on each person’s table, with the benchmark rice in the center and the other three around it. This arrangement was to avoid unwittingly introducing “lining up bias” regarding one rice type being viewed as having a higher quality had they been presented linearly (e.g., see Demont et al., 2012). In line with dominant preferences for broken rice, confirmed by Table 1, all four rice products complied with the “100% broken grain” market standard. Here, it is instructive to note that preferences for broken versus unbroken rice are heterogeneous among consumers, despite the dominance of the former. Thus, an important advantage of keeping the “100% broken grain” standard constant across the four rice types was the avoidance of endogeneity, which enabled further analysis based on preferences for broken versus unbroken rice. In the experiment, it was explained that the visual and sensory characteristics of the uncooked rice could be examined over the course of two bidding rounds separated by a tasting session. It was also mentioned that only one product and one round would be binding.

We followed Haines et al. (1988) and used a dual approach, which first requested participants to choose between the benchmark rice and each of the alternative rice versions, selected in a random fashion by the enumerator. For those who chose the benchmark, a value of zero was recorded; for those who preferred the upgrade, WTP for upgrading was elicited. This procedure was repeated after a sensory session during which participants were presented with cooked versions of each rice type, which enabled them to experience the sensory characteristics of the cooked rice for each of the three options. This included the aroma, texture, swelling capacity, and taste of the cooked rice. In order not to taint their sensory perceptions, participants were requested to cleanse their palates with water in between tasting. Between the pre-sensory and post-sensory rounds, a survey was administered to collect socio-demographic information. A second-round survey was administered after the post-sensory round to gather data on participants’ rice preferences and other characteristics such as their attitudes, purchasing habits, and awareness of NERICA. Upon completing the surveys, one round and one product was chosen at random, at which point the highest bidder received the local rice auctioned in the chosen round. All participants were then paid and thanked for their time, with the highest bidder receiving their participation fee less the bid of the second highest bidder.

## Data and descriptive statistics

Variables used in modeling WTP including demographic variables are shown in Table 1. About 78% of participants were reportedly aware of NERICAs, which was in stark contrast with Uganda where only a third of participants knew about them (Britwum et al., 2020). As noted, NERICA was widely promoted in The Gambia during its introduction in the early 2000s on both radio and television (Reece et al., 2011). While 14% of participants were from households that purchased rice daily, nearly half of them purchased rice monthly. In terms of purchase habits which we hypothesize to be induced by colonial heritage, 85% of the participants expressed clear preferences for broken rice, justifying our choice to keep the “100% broken grains” market standard constant among the four rice products. In addition, 26% of the respondents mentioned swelling capacity as their number one criterion for judging rice quality. Both indicators suggest that while colonial heritage has had a dominant influence on preferences, some heterogeneity among consumers remains, which can help disentangle the effect of colonial heritage on WTP.

**Table 1.** Variable definition and descriptive statistics.

Variable	Description	Mean (Std Dev.)
*Experimental procedure*		
Morning	1 if field experiment was conducted in the morning; 0 otherwise	0.50 (0.50)
Hungry	1 if subject self-reported to be hungry during the session; 0 otherwise	0.38 (0.49)
*Attitudes and knowledge*		
Awareness of NERICA	1 if subject was aware of NERICA prior to the experiment; 0 otherwise	0.78 (0.41)
Per capita consumption	Annual quantity of rice consumed per capita (kg)	97.60 (35.65)
Daily purchase	1 if household usually purchases rice on a daily basis; 0 otherwise	0.14 (0.35)
Monthly purchase	1 if household usually purchases rice on a monthly basis; 0 otherwise	0.49 (0.50)
*Purchase habits*		
Swelling capacity	1 if subject mentions swelling capacity as first criterion in judging rice quality	0.26 (0.44)
Preference for broken rice	1 if subject prefers 100% broken rice over unbroken rice; 0 otherwise	0.85 (0.36)
*Demographics*		
Cultural heritage	1 if subject is genealogically related to domesticators of rice; 0 otherwise	0.62 (0.49)
Cooking housemaid	1 if household has a cooking housemaid; 0 otherwise	0.08 (0.27)
Trader	1 if subject is active in trading; 0 otherwise	0.46 (0.50)
Housewife	1 if subject is a housewife; 0 otherwise	0.11 (0.31)
Group membership	1 if subject is member of a group; 0 otherwise	0.59 (0.49)
Age	Subject age in years	30.95 (9.25)
Higher education	1 if subject has at least secondary or tertiary education; 0 otherwise	0.48 (0.50)
Family income	Average monthly household income in 1,000 Gambian Dalasi	3.11 (1.68)
Household size	Number of individuals in the household	10.03 (5.64)

*Note*: The exchange rate at the time of the auctions was US$1 = GMD 30.55.

More than half of participants, specifically 62% of them, were from ethnicities traced to original domesticators of African rice species. These were *Mandinka, Jola, Karoninka, Serahule, Manjago,* and *Temne*. These ethnicities can be traced back to the indigenous peoples in Mali, Casamance in Senegal, Guinea, and Guinea Bissau who were early domesticators of the *O. glaberrima* species. The *Mandinka* are descendants of the *Mandé* people, who together with the *Jola* are known to have originally domesticated the African rice varieties. The remaining groups, *Karoninka, Serahule, Manjago,* and *Temne*, had a prolonged exposure with the *Mandé* and *Jola* ethnic groups. Thus, they were all considered to have a rich rice cultural heritage, with lineages traced to the early domesticators. Participants whose ancestry were traced to *Bambara, Wolof, Serere*, *Fula*, and *Aku* ethnicities were considered “immigrants,” with lineages traced to peoples who settled in The Gambia from elsewhere, or were traditionally engaged in livestock or cultivation of crops other than rice.

We followed Haines et al. (1988) and modeled decisions of participants in the experiments as a two-stage process, participants’ desirability to upgrade, and their WTP. Further details about modeling and hypotheses for the variables are included in a supplementary material.

## Results

Descriptive statistics of the 2010 dataset were captured in Demont et al. (2013a) and Demont and Ndour (2015). Here, we generate more in-depth insights by analyzing the data through a more formal econometric approach. Prior to examining the model’s results, we present a summary of participants’ WTP bids across pre-sensory and post-sensory rounds, displayed in Table 2. Also shown is the proportion of zero bids. Given that bids were submitted to upgrade the benchmark rice to each of the three local alternatives (Peking rice, unlabeled NERICA, and labeled NERICA), these amounts are also considered price premiums. For both pre- and post-sensory rounds, the lowest bids were submitted for upgrading to one kilogram of Peking rice. Incidentally, Peking rice also recorded the highest proportion of zero bids; half of all bids submitted in the post-sensory round. The higher premium bids were recorded for the NERICA variety, and especially so for the labeled option in both rounds. The fact that the highest price premiums were given for the labeled NERICA option suggests that NERICA brand equity among consumers was strong.

**Table 2.** Descriptive statistics of WTP bids (in Gambian Dalasi, GMD).

	Peking rice		Unlabeled NERICA		Labeled NERICA	
	% zero bids	Mean bid	% zero bids	Mean bid	% zero bids	Mean bid
Pre-sensory	42%	2.70	33%	3.39	16%	4.09
Post-sensory	50%	2.25	32%	2.62	23%	3.56

*Note*: The exchange rate at the time of the auctions was US$1 = GMD 30.55.

### Results from the double-hurdle model

Summarized in Table 3 are results from the double-hurdle model for both participation and purchase equations. A total of 600 observations were generated across the two experimental rounds and three rice products for the 100 participants. Due to missing observations, the model was estimated for 558 of the 600 initially generated observations. As there were multiple observations for each participant across rounds and products, the model was cluster corrected, and reported standard errors are robust.

**Table 3.** Results of double-hurdle model: propensity of upgrading and willingness to pay.

	Tier 1: Participation equation	Tier 2: Purchase equation
Parameter	Coefficient (SE)	Coefficient (SE)
Constant	0.625 (0.705)	0.261 (5.352)
Post-sensory	−0.151 (0.110)	−0.018 (0.054)
NERICA intrinsic^a^	0.350 (0.132)***	−0.236 (0.220)
NERICA extrinsic^b^	0.493 (0.116)***	1.337 (0.348)***
Morning	−0.512 (0.186)***	−2.017 (2.239)
Hungry	0.226 (0.190)	−0.064 (1.132)
Awareness of NERICA	0.323 (0.224)	−1.419 (2.087)
Per capita consumption	−0.001 (0.002)	−0.006 (0.014)
Daily purchase	−0.293 (0.315)	−4.548 (2.789)
Monthly purchase	−0.096 (0.226)	0.218 (1.721)
Swelling capacity	−0.407 (0.207)**	1.166 (1.605)
Preference for broken rice	−0.730 (0.391)*	11.072 (5.012)**
Cultural heritage	−1.515 (0.427)***	5.802 (3.354)*
Cultural heritage × preference for broken rice	1.260 (0.486)***	−9.661 (4.742)**
Cooking housemaid	0.279 (0.257)	7.648 (2.340)***
Trader	−0.142 (0.254)	−3.057 (1.702)*
Housewife	0.207 (0.274)	−2.432 (2.251)
Group membership	0.100 (0.178)	−1.094 (2.395)
Age	0.018 (0.011)	0.142 (0.098)
Higher education	−0.115 (0.197)	−1.144 (2.253)
Family income^c^		−2.719 (2.581)
Family income squared^c^		−0.673 (0.506)
Household size	0.012 (0.016)	0.152 (0.146)
*Variance: Constant*		−1.066 (0.267)***
*Variance: Family income*		2.640. (0.498)***

*Notes*: The exchange rate at the time of the auctions was US$1 = GMD 30.55. Number of observations = 558; significance levels: *p < 0.1, **p < 0.05, ***p < 0.01. Standard errors (SE) are robust and cluster-corrected. ^a^This dummy captures both unlabeled and labeled NERICA relative to Peking rice, the reference dummy. ^b^This dummy captures labeled NERICA relative to unlabeled NERICA and Peking rice. ^c^Household income was not included in Tier 1, which only contains non-economic variables (Britwum et al., 2020; Dong et al., 2004; Newman et al., 2003).

Beginning with the rice versions, the locally grown Asian benchmark Peking was designated the reference in the product category, with two incremental dummy variables created for NERICA, as explained in the modeling section (see supplementary material). NERICA generally proved popular among respondents, relative to Peking rice. When confronted with the intrinsic quality attributes of NERICA (labeled and unlabeled), participants were found to be more likely to upgrade the imported *Bella Rosa* rice to NERICA, than to Peking rice. However, the NERICA label further boosted NERICA’s market share relative to Peking rice. This points to both the intrinsic value of NERICA and the effectiveness of the extensive media coverage of the variety (Reece et al., 2011) in The Gambia. Preferences for NERICA were further exemplified by participants’ willingness to pay price premiums for them. Although NERICA’s intrinsic value was not significantly different from Peking rice, the NERICA label attracted a price premium.

The *morning* variable was statistically significant; those who participated in the auctions in the afternoons were more likely to upgrade to the alternative local rice varieties, which contrasts with previous findings in Benin, Senegal, and Uganda (Britwum et al., 2020; Demont et al., 2012, 2013b, 2013c). While further studies would be helpful in further explaining this phenomenon, time of auctions have on occasions not yielded any significant impacts on preferences and WTP (e.g., Demont et al., 2017; Diagne et al., 2017).

The variables *cultural heritage*, on the one hand, and *preference for broken rice* and *swelling capacity* on the other hand are assumed to be suitable proxies for assessing whether participants’ preferences for rice have been influenced by cultural or colonial heritage, respectively. Consumers with cultural heritage were found to be less likely to upgrade the benchmark, but when they did, paid higher price premiums for the upgrades. Consumers who prefer broken rice with high swelling capacity were more likely to “keep” the imported benchmark instead of exchanging it into any of the local alternatives. However, those who decided to upgrade their imported benchmark to local varieties were willing to pay a price premium for the latter. The interaction term between *cultural heritage* and *preference for broken rice* is interesting. Its coefficients suggest that participants with genealogical linkages to early rice domesticators, but whose preferences have been altered by colonial heritage (from unbroken to broken rice) were more likely to upgrade the benchmark, but paid lower price premiums for the upgrades.

The remainder of the results showed that demographics generally had a weak impact on preferences for local rice. Specifically, only two demographic variables were statistically significant in the purchase equation in Table 3. Although household income was not statistically significant, a close proxy for income, cooking housemaid, was. Households with a cooking housemaid tend to be wealthier, or are likely to have higher opportunity costs of time. Similar to findings in Senegal (Diagne et al., 2017), we observed that participants who employed cooking housemaids at the time of the experiment were willing to pay higher price premiums for the local varieties. Similar to previous findings in Saint Louis, Senegal (Demont et al., 2013b), traders tended to offer lower price premiums for exchanging imported rice into the local upgrades. This could be because they are likely more aware of available substitutes on the market or because they are professionally more experienced with “starting low” price bargaining (Canavari et al., 2019). The household income variable was significant in the variance portion of the model, an indication of wide income variability among participants.

To interpret the results in Table 3 in a meaningful way, in Tables 4 and 5, we categorized Gambian rice consumers into four segments. As Tynan and Drayton (1987) noted, a number of reasons underscore consumer segmentation including geography and demographics. Although our segmentation is largely motivated by these two factors, this analysis is posterior to the experiments, as they were conducted well before the cultural heritage concept was first proposed and validated. For this study, the segments were specifically based on consumers’ heritage as evidenced by their preferences and genealogical lineages. The segments were: (i) rice consumers with cultural heritage who also feature preferences induced by colonial heritage (Segment 1); (ii) rice consumers without cultural heritage but featuring preferences induced by colonial heritage (Segment 2); (iii) rice consumers with cultural heritage and featuring preferences unaltered by colonial heritage (Segment 3); and (iv) rice consumers without cultural heritage and featuring preferences unaltered by colonial heritage (Segment 4).^1^ Table 4 provides additional demographic background in each of the four segments. Awareness of NERICA was generally high, but was slightly lower in Segment 2 than the overall average. Participants in Segment 2 were, however, more likely to be traders, with approximately 63% having at least had a secondary school education, the highest among the four segments. These are likely indicative of the urbanized group who had preferences induced by colonial heritage. Participants in Segment 3 on average had the lowest levels of education, but with larger household sizes. Those whose preferences were induced solely by colonial heritage, and those with unadulterated indigenous preferences recorded similarly high levels of consumption per capita, with segments 1 and 4 with lower consumption levels.

**Table 4.** Descriptive statistics of segments.

	Mean (Standard deviation)				
	All	Segment 1	Segment 2	Segment 3	Segment 4
Awareness of NERICA	0.78 (0.41)	0.83 (0.38)	0.69 (0.46)	0.78 (0.42)	0.83 (0.38)
Per capita consumption	97.60 (35.65)	93.31 (30.98)	103.50 (33.85)	103.93 (61.56)	94.55 (25.19)
Trader	0.46 (0.50)	0.42 (0.49)	0.56 (0.50)	0.44 (0.50)	0.33 (0.48)
Higher education	0.48 (0.50)	0.45 (0.50)	0.63 (0.49)	0.22 (0.42)	0.33 (0.48)
Household size	10.03 (5.64)	10.62 (6.33)	8.78 (4.21)	11.50 (6.22)	9.17 (3.33)

**Table 5.** Quality competitiveness of NERICA relative to imported *Bella Rosa* as revealed through its incremental intrinsic and total (intrinsic and extrinsic) value per kilogram in four market segments induced by cultural and colonial heritage.

Cultural heritage		Yes	No
Colonial heritage	Yes	**Segment 1** Variable: Cultural heritage × preference broken Intrinsic price premium = GMD 1.73 (12%) Total price premium = GMD 2.73 (20%) Population share = 54%	**Segment 2** Variable: Preference broken Intrinsic price premium = GMD 3.02 (22%) Total price premium = GMD 4.01 (29%) Population share = 32%
	No	**Segment 3** Variable: Cultural heritage Intrinsic price premium = GMD 0.67 (5%) Total price premium = GMD 1.67 (12%) Population share = 9%	**Segment 4** Variable: Reference Intrinsic price premium = GMD 1.33 (10%) Total price premium = GMD 2.33 (17%) Population share = 5%

*Notes*: The exchange rate at the time of the auctions was US$1 = GMD 30.55. Price premiums are computed based on unconditional partial effects (Equation 7 in Burke, 2009). Price premiums in percentage are computed by dividing the price premium in GMD by the price of the benchmark (GMD 14/kg).

For meaningful comparisons to be drawn between these segments beyond their demographics, we computed price premiums based on unconditional partial effects (see equation 7 in Burke, 2009) derived from our econometric results in Table 3. As the *cultural heritage* and *preference for broken rice* variables were in both tiers of the double-hurdle model, this approach enabled us synthesize participants’ decisions in both tiers through a single value metric. Price premiums were estimated for intrinsic and total values of NERICA. While the intrinsic price premiums captured the value of labeled and unlabeled NERICA, total price premiums captured the value of intrinsic and extrinsic attributes of NERICA. In addition to expressing the premiums as percentages over the benchmark *Bella Rosa* rice, the population shares of these market segments were also estimated.

## Discussion

Gauging from the estimated price premiums in Table 5, preferences for 100% broken NERICA in The Gambia can be ranked across the four consumer segments, beginning with those with only colonial heritage (Segment 2), followed by those with preferences straddling both cultural and colonial heritage (Segment 1), then to those whose preferences have been influenced by neither (Segment 4), and finally those with cultural heritage and featuring preferences unaltered by colonial heritage (Segment 3). Consumers in Segment 3 were willing to pay the lowest price premiums, i.e. between GMD0.67 to GMD1.67 per kilogram (US¢2.19–5.47) or 5–12% of the price of *Bella Rosa*. Since this market segment has preferences for unbroken rice, none of the rice types (all of them 100% broken) may have appealed to them, leading to a low overall involvement in the auction and low interest in trading a non-preferred benchmark to a non-preferred alternative. However, when we omit cultural heritage and move from Segment 3 to Segment 4, price premiums increase by 5 percentage points (i.e., GMD1.33–2.33 [US¢4.35–7.63] or 10–17% of the benchmark price). With the introduction of colonial heritage, that is, in moving from Segment 3 to Segment 1, instead, price premiums rise by about 7 percentage points (i.e., GMD1.73–2.73 [US¢5.66–8.94] or 12–20% of the benchmark price). Finally, when we remove cultural heritage and move from Segment 1 to Segment 2, price premiums further increase by about 10 percentage points (i.e., GMD3.02–4.01 [US¢9.89–13.13] or 22% to 29% of the benchmark price). Incidentally, participants in Segment 2 were the least aware about NERICA, at 69%. Cultural and colonial heritage clearly have opposing effects on price premiums for NERICA relative to the imported Asian benchmark; cultural heritage erodes price premiums by 5–10 percentage points, while colonial heritage boosts price premiums by 7–12 percentage points. The question that remains is the extent to which these results have been generated by our experiment’s focus on the “100% broken” grain quality standard, which is generally more aligned with preferences induced by colonial heritage than with indigenous preferences of the inheritors of cultural heritage. Future research needs to be conducted in which consumers’ WTP for broken and unbroken rice are compared and correlated with their heritage.

Equally noteworthy are the population shares across the four market segments which indicate that the majority of consumers have preferences induced by both colonial and cultural heritage. Those with cultural heritage and preferences unaltered by colonial heritage make up less than 10% of the sample, suggesting substantial shifts toward colonial heritage-induced preferences likely attributable to import substitution policies over the decades. Given the relatively small sample size, however, our findings may not be generalizable across The Gambia, and we note this as a limitation. Similarly, since the experimental auctions were conducted before the initial hypothesis of cultural heritage, the four segments may not adequately reflect consumers’ backgrounds. It should be noted though, that the goal of segmentation was not to show significant differences in price premiums between them, but rather, to determine whether there were positive price premiums for NERICA among consumers whose preferences have been influenced (or not) by cultural and/or colonial heritage.

Our findings are insightful for a couple of reasons. First, they show that agronomic superiority of local rice varieties by itself may not be enough if the goal is to improve market shares relative to imported rice. The segmentation of consumers matters, and our findings show it is crucial that new varieties are tailored to meet consumers’ varying preferences. Second, although NERICA was originally bred to embody superior characteristics from both Asian and African varieties—that is, the high yielding characteristics of the former and the disease and drought resistance of the latter—the blend of the varieties have yielded traits that appear to mesh well with the dual consumer preferences induced by cultural and colonial heritage. As these findings suggest, the target market for NERICA is consumers whose trait preferences have either been influenced by both cultural and colonial heritage, or predominantly by colonial heritage. Together, they constitute approximately 86% of consumers, and as our results show, they are willing to pay price premiums between 12% to 29% over the Asian import, *Bella Rosa*. These high WTP price premiums for NERICA should be viewed as encouraging to breeders, farmers, value chain stakeholders, and policymakers in The Gambia. In addition to being cognizant of consumer compositions, recent findings from Senegal have shown that consumers prefer rice products with a quality finish such as grain homogeneity, cleanliness, and products rid of impurities (Mané et al., 2021). For The Gambia, this would warrant investments across various segments of rice value chain development, particularly toward modern industrial and semi-industrial milling technologies (Soullier et al., 2020).

Finally, since the data was collected just after the introduction, adoption, and widespread promotion of NERICA in The Gambia, it reflects a snapshot of pure demand at an important momentum, i.e. before NERICAs got mainstreamed and commingled with other local varieties on the market. In Uganda, this challenge of comingled NERICA with third varieties prompted a study by Britwum et al. (2020) who undertook a retrospective look at NERICA varieties to draw important lessons for trait prioritization in breeding. It was argued in the study that “Similar retrospective studies need to be conducted in other countries…” (p. 308). Moreover, following the initial, strong validation of the cultural heritage hypothesis (Demont et al., 2017), new opportunities for revisiting datasets collected prior to this research thread have opened up. Both arguments informed the retrospective approach adopted in this study. However, as the main focus of our study was to assess how cultural and colonial heritage affect consumer preferences and breeding priorities, we believe that our findings contribute useful insights to this research thread and are relatively time invariant.

## Conclusion

Lessons across the continent about value chain improvements consistently show that emphasis on productivity gains alone could hamper adoption goals if new varieties are not tailored to consumer preferences. In The Gambia, large investments in irrigation which largely overlooked the cultural dynamics of communities they were intended to benefit proved costly, deviating markedly from anticipated productivity goals in rice production. Toward understanding factors that shape Gambian consumers’ attitudes toward rice, this study used auction experiments to examine preferences for local rice varieties, a New Rice for Africa (NERICA) variety, and another local version, Peking rice. The specific goal was to examine rice preferences between consumers endowed with centuries-long cultural heritage as a result of their lineage or proximity to centers of early rice domestication, and consumers outside the cultural heritage sphere (referred in the study as having preferences induced by colonial heritage). Either of these, cultural heritage, or colonial heritage represent different rice attribute preferences; unbroken grains with no fragrance for the former, and broken grains, fragrance and higher swelling capacity for the latter.

Study findings show that NERICA proved popular, fetching significantly higher price premiums than Peking rice. Consumers with both cultural and colonial heritage were willing to pay price premiums for NERICA relative to the inferior imported Asian rice, with higher premiums among those whose preferences were influenced solely by colonial heritage. This is suggestive of the broken grain standard resonating with colonial heritage-induced preferences.

These results offer new perspectives for breeders, agronomists, policy makers and value chain actors. For breeders, it is imperative both cultural and colonial heritage rice traits underpin the development of new varieties to broaden their appeal to diverse consumer segments. Policies that encourage the development of new varieties could also draw lessons from the media promotion of NERICA in The Gambia and its subsequent popularity among farmers. Ultimately, for rice self-sufficiency goals to be successful, they should extend beyond agronomic traits, and incorporate preferences suited to consumers whose preferences have been shaped by cultural heritage, induced by colonial heritage, or both.

## Supplemental Material

Supplemental Material, sj-pdf-1-oag-10.1177_00307270211019758 - Tailoring rice varieties to consumer preferences induced by cultural and colonial heritage: Lessons from New Rice for Africa (NERICA) in The Gambia Click here for additional data file. Supplemental Material, sj-pdf-1-oag-10.1177_00307270211019758 for Tailoring rice varieties to consumer preferences induced by cultural and colonial heritage: Lessons from New Rice for Africa (NERICA) in The Gambia by Kofi Britwum and Matty Demont in Outlook on Agriculture

## Note

1. As a robustness check, the model for Table 3 was run without the cultural heritage x preference for broken rice interaction variable. Instead, three dummy variables were created, such that Segment 1 = 1 if colonial heritage = 1 and cultural heritage = 1; 0 otherwise; Segment 2 = 1 if colonial heritage = 1 and cultural heritage = 0; 0 otherwise; Segment 3 = 1 if colonial heritage = 0 and cultural heritage = 1; 0 otherwise. Segment 4 served as the reference category. Estimated price premiums were exactly the same as displayed in Table 5. See supplementary material.
