# Supplementary material for: Tailoring rice varieties to consumer preferences induced by cultural and colonial heritage: Lessons from New Rice for Africa (NERICA) in The Gambia
Source: Outlook Agric. 2021 May 26;50(3):305–14. doi: 10.1177/00307270211019758 (PMC8493303; doi:10.1177/00307270211019758)
Supplement: Supplemental Material, sj-pdf-1-oag-10.1177_00307270211019758 - Tailoring rice varieties to consumer preferences induced by cultural and colonial heritage: Lessons from New Rice for Africa (NERICA) in The Gambia [file sj-pdf-1-oag-10.1177_00307270211019758.pdf]

Econometric Model

We followed Haines et al. (1988) and modeled decisions of participants in the experiments as a two-stage process. The first stage related the participant's desirability to upgrade, and the second stage their WTP; that is, how much the participant bid to upgrade from the benchmark to the alternative. These decisions were modeled using the double-hurdle approach (Cragg 1971) where different stochastic processes are used for a participant's willingness to upgrade, and their WTP. Further computational details are explained in Burke (2009).

Assume d_{ijpr} represents participant i's desirability to upgrade one kilogram of the benchmark rice for an alternative rice p, in session j and bidding round r, and WTP_{ijpr} is the amount a participant is willing to spend as captured by their submitted bids. Then,

$$d_{ijpr} = 1 \text{ if } \boldsymbol{Z}_{ijpr}\delta + u_{ijpr} > 0$$

$$d_{ijpr} = 0 \text{ if } \boldsymbol{Z}_{ijpr}\delta + u_{ijpr} \leq 0 \quad (1)$$

$$WTP_{ijpr} = \boldsymbol{X}_{ijpr}\beta + \varepsilon_{ijpr} \quad (2)$$

where $\boldsymbol{Z}$ is a vector of explanatory variables that explain the desirability to upgrade, $\boldsymbol{X}$ a vector of variables that determine WTP, and δ and β vectors of parameters to be estimated. The error terms, u_{ijpr} and ε_{ijpr}, are assumed to be normally distributed with variance equal to 1 and σ^2, respectively. A participant's desirability to upgrade, d_{ijpr}, is a binary indicator which is equal to 1 if WTP is positive, and 0 otherwise. The probability a respondent decides not to submit a positive bid can be expressed as:

$$\Pr\left(WTP_{ijpr} = 0 \middle| \boldsymbol{Z}_{ijpr}, \boldsymbol{X}_{ijpr}\right) = \Phi\left(-\boldsymbol{X}_{ijpr}\beta/\sigma\right) + \Phi\left(\boldsymbol{X}_{ijpr}\beta/\sigma\right)\Phi\left(-\boldsymbol{Z}_{ijpr}\,\delta\right) \quad (3)$$

The distribution of WTP being positive is given as:

$$f\left(WTP_{ijpr}|\boldsymbol{Z}_{ijpr}, \boldsymbol{X}_{ijpr}, WTP_{ijpr} > 0\right)$$

$$= (2\pi)^{-1/2}\sigma^{-1}\, exp\left\{-\,(WTP_{ijpr} - \boldsymbol{X}_{ijpr}\beta)/2\sigma^2\right\}\Phi\left(-\boldsymbol{Z}_{ijpr}\,\delta\right) \qquad (4)$$

where Φ is the standard normal cumulative density function.

To capture the “two” NERICA versions (labeled and unlabeled) used in the auctions, two incremental product dummy variables were created. First, a NERICA dummy equal to 1 was constructed for both the labeled and unlabeled product. This captures the intrinsic value of NERICA (*O. glaberrima* × *O. sativa* cross), relative to Peking rice (*O. sativa*). Next, to capture the extrinsic value of NERICA, an incremental dummy was constructed, equaling 1 whenever the NERICA label appeared on the bag corresponding to the product and 0 otherwise. The goal here was to disentangle the extrinsic from the intrinsic value of NERICA, relative to the most popular locally grown Asian variety in the region and the Asian import product. Finally, to control for possible heteroscedasticity in our model, all variables in the WTP equation were tested for heteroscedasticity, with those that emerged significant left in the variance portion of the model.

Hypotheses

Hypotheses about the model’s coefficients and their expected signs were largely informed by findings from the literature on rice preferences among SSA consumers. Beginning with the products, previous studies have found positive consumer responses to rice brands signaling local origin (e.g., Costello et al. 2013; Demont et al. 2013a, b). Thus, it was anticipated that the propensity to exchange the inferior benchmark and WTP price premiums would be higher for the labeled NERICA product than the unlabeled NERICA and Peking products. Awareness of NERICAs was expected to boost preferences and WTP, particularly for the labeled product. In terms of the broken grains trait, very little has been done to gauge preferences for this characteristic

across different rice offerings. Although broken rice has been popularized as a result of colonial import substitution policies, it was not known with certainty whether preference for broken rice would translate into preferences for or against the local rice varieties in the experiments, given that all rice products complied with the “100% broken” standard.

For experimental procedure, the influence of the time of auctions was deemed uncertain. While some previous studies have found stronger preferences and high WTP premiums in the morning (Demont et al. 2012; Demont et al. 2013a, b), others have found that time of auctions did not matter, and had little significant impacts on preferences and WTP (Diagne et al. 2017; Demont et al. 2017). Regarding the former, it has been explained that subjects who participate in auctions prior to their purchasing rice in the markets (i.e., in the mornings) tend to value the products higher than those who may have already purchased rice prior to their participation in the experiments.

With respect to demographics, participants with cooking housemaids were expected to more willingly upgrade and pay price premiums for the alternative rice (similar to Diagne et al. 2017), although other studies have found lower preferences among such consumers (Britwum et al. 2020; Demont et al. 2012). Demont et al. (2013a) found that participants in rice auctions in Saint-Louis, Senegal, who were also traders were less likely to exchange a benchmark rice for the alternative. Given the close proximity of The Gambia with Senegal, similar outcomes were anticipated among traders. A key demographic variable in this study is *cultural heritage*. Studies that have validated the cultural heritage hypothesis all point to strong preferences and WTP for local rice among consumers with lineages to early rice domesticators (Demont 2013; Demont and Ndour 2015; Demont et al. 2017). However, given that we standardized the grain quality of all four rice products in the experiment to 100% broken grains, an “imported” market standard heavily influenced by the introduction of Asian rice in the Senegambia region (John 2015), it was difficult

to anticipate how consumers would trade off preferences induced by cultural versus colonial heritage into their valuation of the local and imported rice products. This was a strong justification for our study.

Results

We present an alternative to the study's results in Table 3 of the main manuscript. In this alternative as show in Table 1 below, dummy variables for consumers segments as induced by cultural and/or colonial heritage are constructed. Segment 1 represents consumers with preferences induced by both cultural and colonial heritage, Segment 2 for consumers with preferences induced by colonial heritage and not altered by cultural heritage, and Segment 3 for consumers with cultural heritage only. Consumers with preferences induced by neither cultural nor colonial heritage were designated as the reference group. This approach provides the same results and price premium outcomes as shown in the manuscript.

Table 1. Results of double-hurdle model: Propensity of upgrading and Willingness to pay

Parameter	Tier 1: Participation equation Coefficient (SE)	Tier 2: Purchase equation Coefficient (SE)
Constant	0.625 (0.705)	0.261 (5.352)
Post-sensory	–0.151 (0.110)	–0.018 (0.054)
NERICA intrinsic [a]	0.350 (0.132)***	–0.236 (0.220)
NERICA extrinsic [b]	0.493 (0.116)***	1.337 (0.348)***
Morning	–0.512 (0.186)***	–2.017 (2.239)
Hungry	0.226 (0.190)	–0.064 (1.132)
Awareness of NERICA	0.323 (0.224)	–1.419 (2.087)
Per capita consumption	–0.001 (0.002)	–0.006 (0.014)
Daily purchase	–0.293 (0.315)	–4.548 (2.789)
Monthly purchase	–0.096 (0.226)	0.218 (1.721)
Swelling capacity	–0.407 (0.207)**	1.166 (1.605)
Segment 1	-0.986 (0.352)***	7.213 (3.408)**
Segment 2	–0.730 (0.391)*	11.072 (5.012)**
Segment 3	–1.515 (0.427)***	5.802 (3.354)*
Cooking housemaid	0.279 (0.257)	7.648 (2.340)***
Trader	–0.142 (0.254)	–3.057 (1.702)*
Housewife	0.207 (0.274)	–2.432 (2.251)
Group membership	0.100 (0.178)	–1.094 (2.395)
Age	0.018 (0.011)	0.142 (0.098)
Higher education	–0.115 (0.197)	–1.144 (2.253)

Family income [c]		–2.719 (2.581)
Family income squared [c]		–0.673 (0.506)
Household size	0.012 (0.016)	0.152 (0.146)
Variance: Constant		–1.066 (0.267)***
Variance: Family income		2.640. (0.498)***
